# A conversation analytic approach to *schizophrenic* interaction: methodological reflections on disruptions of the common-sense world

**DOI:** 10.3389/fsoc.2023.1223186

**Published:** 2023-12-19

**Authors:** R. G. Smith, Lesley Stirling

**Affiliations:** School of Languages and Linguistics, The University of Melbourne, Melbourne, VIC, Australia

**Keywords:** schizophrenia, conversation analysis, common-sense, phenomenology, ethnomethodology, talk-in-interaction, multiple realities

## Abstract

Certain schools of phenomenological psychiatry conceive of *schizophrenia* as a pathology of common-sense. Ethnomethodological enquiry, with its roots in Schutzian social phenomenology, takes as its domain, topic, and substance of study the ongoing achievement of a common-sense world between social members. Yet, dialogue between psychiatry and ethnomethodological approaches is thin. In this article, we discuss a conversation analytic approach to *schizophrenic* interaction which has generated and utilized a model of a five-world manifold to frame analyses of talk-in-interaction. ‘Worlds’ are conceived, after Schutz, as finite domains of meaning, and the model operates as a breach of natural attitude assumptions to examine mechanisms of the constitution of the one-world-in-common of common-sense. It is suggested that certain aspects of *schizophrenic* talk might receive account in terms of a loss of integration between these five domains of meaning. Conversation Analytic methods were applied to transcripts of audio recordings of psychiatric interviews but encountered hurdles that motivated the broadening of methodological scope. Such hurdles included a weakening of the next turn proof procedure, implicit reification of the *schizophrenia* construct, and problems of translation presented by the analyst’s normative membership encountering non-normative life-worlds of *schizophrenic* experience. Strategic responses to these hurdles included exploring linkages between phenomenological psychiatry and ethnomethodological approaches, as well as an engagement of ethnomethodological self-reflection and conceptual clarification of the *schizophrenia* construct in line with Garfinkel’s unique adequacy requirement. The manifold model is glossed, and interaction between two of its worlds – a world of concrete, situational immediacies and another of abstract organizations – is explored in more detail via analysis of conversational data. It is suggested that the five-world model, along with further micro-analysis of talk-in-interaction, might have implications in psychiatry for topics such as autism, double bookkeeping, concretism, theories of disturbed indexicality, and insight attribution. We conclude that the consideration of atypical interaction obliges the interaction analyst to take account of their own implicit normative world-frames and that the use of domain-specific top-down models in conjunction with the inductive approach of Conversation Analysis may extend the reach of CA to facilitate productive dialogue with other disciplines.

## 1 Introduction

We live in a time of divergent realities. In one version of world events, a heroic mobilization of science appears to have delivered pandemic-stemming vaccines; in other versions, these injections are means to inject computer chips into a docile populous, with, for some, these efforts being driven by shape-shifting reptiles from other dimensions. People who insist upon the latter scenarios, we are told, are not necessarily ‘mad’, and yet these are precisely the types of stories that people who are diagnosably delusional might tell with a conviction that seems to transcend mere belief.

In this article, we discuss a conversation analytic approach to *schizophrenic*^1^ interaction which has generated and utilized a model of a five-world manifold to frame analyses of talk-in-interaction. It is born out of such questions as how it might be that people come to inhabit such differing versions of reality and, to the extent that they do inhabit them, how it is they might keep one foot sufficiently in a common reality to navigate social organizations with radically different underlying world-structure. Perhaps most bafflingly, we have been driven to enquire how it is that some people who perform this feat are deemed diagnosably delusional, while others are not.

We have not, by any stretch of the imagination, answered any of these motivating questions; rather, the process of enquiry has led us, ultimately, to question how ‘normal’ social members come to inhabit common realities in the first place.

The Oxford neuroscientist Anil Seth (2021) presents one version of this question: Current models of brain function suggest that the fundamental task of the brain is prediction, and the brain does this by generating plausible models that are then measured against external input and tweaked according to this feedback. The predictive models in themselves *are* our experience of the world, progressively shaped by the external input. In effect, our experience of ‘reality’ is active, projective hallucination. There is no ‘light’ beneath the skull, no ‘sound’, no ‘touch’, only neuro-electric activity. Our experiences of ‘light’, ‘sound’, and ‘touch’ are grounded in, but distinct from, these patterns of neural impulse within the silent darkness of the perpetually solipsistic skull. The world, as we hallucinate it, is an extremely private affair—so how is it that we come to ‘share’ a world in the first place? How do we come to coordinate and mutually inhabit our world hallucinations so effectively?

Putting the question in this way, of course, puts the riddle of hallucination in *schizophrenia* – most commonly, the hearing of voices that other people *do not* hear – entirely on the other foot. It is suddenly *not* so strange that people hallucinate voices – this is a fundamental mechanism of audition – but now other questions arise, such as: When I (a non-*schizophrenic*) hallucinate the voice of my interlocutor, how can I be confident that another nearby auditor will be hallucinating the same thing?

In the end, our strategic response to such questions has been to say: Ok, rather than bringing *schizophrenia* to account against common-sense world conceptions (to justify it in other words, as words are justified against the straight edge of a page), let us instead adjust our world-conceptions to accommodate reports of *schizophrenic* experience, and accept whatever alienating strangeness that might bring us to.

This brief consideration of hallucination exposes ‘common-sense’ as having two faces: ‘sense-as-perception’ (held in common) and ‘sense-as-meaning’. Certain approaches to phenomenological psychiatry conceptualize *schizophrenia* as a pathology of common-sense or a disturbance in the ground of ‘self’ in micro-social intersubjectivity (Blankenburg and Mishara, 2001; Stanghellini, 2004; Phillips, 2019). The motivating question for this study has been whether any such disturbance of common sense organization might be evidenced through analysis of *schizophrenic* talk-in-interaction. The intended audience for the study includes language and interactional researchers, but also, importantly, psychiatric researchers and clinicians, who, while probably unfamiliar with interactional language research, might nonetheless find its methods relevant and hopefully useful within their own fields.

The initial intention to perform simple bottom-up analysis of conversational data, however, hit methodological hurdles that necessitated familiarization with theory, most notably, in an attempt to forge connections between the theoretical frames of Conversation Analysis (CA) and phenomenological psychiatry. It was found that the simplest way to do this was to conceptualize *schizophrenia* as a ‘world disturbance’ rather than a ‘self-disturbance’. In CA terms, this might be seen as a disturbance in the coordination of settings. This generated the model of a world manifold, with the idea of a ‘manifold’ suggesting independent domains of meaning-organization that coordinate within the common-sense world as a unified domain. The use of such models within Conversation Analysis is, of course, discouraged, yet we suggest it might have implications for psychiatry (or related fields), where understanding the micro-design of talk-in-interaction presents less as an end-in-itself but might nonetheless prove of instrumental interest. Within those interpretive traditions of clinical psychiatry which emphasize ‘understanding’ (*Verstehen*) over ‘explanation’ (*Erklären*)^2^, for instance, effective interpretive models might help facilitate therapeutic engagement and dialogue with patients. In addition, we suggest that the model might hold implications for studying interaction in other atypical populations.

Based on the methodological hurdles this particular study faced and the responses adopted to overcome them, a more general argument is proposed that a dialectic approach might be recommended between domain-specific processes of model construction and bottom-up processes of observation that would otherwise hope to avoid theoretical incursion.

Data for our study of *schizophrenic* talk-in-interaction were drawn from seven audio recordings (no video) of interviews between two interviewers and three female and four male patients who had received a DSM-IV (American Psychiatric Association, 2000) diagnosis on the *schizophrenia* spectrum (diagnoses ranged across schizophrenia, schizophreniform disorder, schizoaffective disorder, and schizotypal personality disorder). These interviews were conducted for an earlier independent study, and the data were made available for the current research. The primary interviewer was a non-treating clinical psychiatrist hoping to pursue language research on *schizophrenia* within a cognitivist frame, and the second interviewer was a psychologist who performed a series of standardized cognitive tests on participants. Interview subjects were recruited from community teams where patients receive treatment under minimal restriction and also from a psychiatric inpatient ward where patient movement is more restricted. All subjects, either in ‘recovery’ or ‘chronic’ phase of psychosis, were receiving regular psychiatric treatment and had English as their first language. All were deemed by regular treating clinicians and the principal psychiatric researcher as competent to consent to participation at the time of the interview, and ethics approval was additionally obtained for the current data analysis. Interviews were conducted in private rooms in either the community or inpatient mental health settings. The interview recordings made available for this study ranged between 21 and 62 min and were all transcribed for the current study following Du Bois (2006) transcription conventions, with local additions.^3^

In this article, in addition to outlining the five-world model that allowed us to get a foothold on the data, we describe a process whereby we came to reflect upon the methodological challenges of applying Conversation Analytic methods to these data and the tactical choices that were required to respond to these challenges.

## 2 Worlds and models

In performing Conversation Analysis, we work a field that was cleared to a significant extent by Harold Garfinkel. But Garfinkel’s ethnomethodology owes an intellectual debt to Alfred Schutz (Heritage, 1984), who himself developed his phenomenological sociology in response and as a complement to the early 20th-century work of Edmund Husserl (Schutz, 1962a). Garfinkel rejected any suggestion that ethnomethodology was a phenomenological method (Garfinkel et al., 1977), but we do not have to agree with him on that – and later, it will be explained why. Certainly, the anti-positivist tenor of the ethnomethodological attitude can be at least partly traced back to Husserl’s critique of scientific objectivism (Moran, 2012). When we get to methods of Conversation Analysis, the pathway back to Husserlian phenomenology is even more overgrown with weeds, and the Husserlian critique of science that lies in CA’s DNA has been largely forgotten.

Husserl provides the first distinction by which we tease apart the different worlds in our model. The broad outlines of his phenomenological method are quite well-known. In daily operations, as well as in most organized practices of science, it is reasonably assumed that the world is simply there, manifestly before us, objectively present. We orient to an external world and not to a world-as-perceived. Husserl describes this underlying assumption as the ‘natural attitude’. However, as we have just suggested, our experience of the world is a somewhat more ‘internal’ affair than this. To avoid what he sees as sterile metaphysical arguments along the lines of a mind/matter divide, Husserl’s first move was to ‘bracket’ questions of ‘external’ reality – to simply put them out of bounds – and to pursue an enquiry into ways the world is revealed within, before, and *as* consciousness. Because the external world (as well as any natural attitude presumptions) has been bracketed, Husserlian phenomenology continues as (world)-transcendent enquiry. There is an inherent critique of scientific objectivity involved here: in seeing the world as an object, the one thing that science cannot take account of is the eye peering down the microscope and the structures, either of consciousness or of mathematical translation, via which the world is revealed. In contrast to the world-as-object, Husserl foregrounded a world-as-experience(d). One inhabits, lives in, and moves through a world that is not a mere object but is shaded by relevance, value, and meaning, a world inherently organized *as* experience, including primordial experiences such as those of threat or danger, habit, habitat, and home, that blur the edges between ‘self’ and ‘world’. Husserl explores these themes of the experienced-world in terms of a ‘life-world’, which is never defined but rather presented as the title of a problem to stimulate enquiry (Moran, 2012, 297). It is not completely clear, for instance, to what extent the life-world represents a world of purely individual experience and to what extent life-world(s?) might be shared. In the case of *schizophrenia,* the distinction between a world that is revealed before the scientific gaze and an experience-imbued lifeworld is the difference between seeing a *diagnosee* through a lens of neurophysiological reduction or as the inhabiter of a lived-world of meanings and values.^4^ The vague outline of two and possibly three different world conceptions can be seen to be emerging here: A ‘world-as-object’, (life)-worlds of private experience, and perhaps (life)-worlds of shared experience.

The question that arose earlier in considering Seth’s model of predictive processing – how to overcome solipsism to arrive at a shared world-in-common – is the same issue that confronted Husserl’s transcendental inquiry. Both models require an account of intersubjectivity. It is at this point that Schutz (1970) diverted his sociological project away from the Husserlian program via an ‘epoché of the natural attitude’. Husserl described the transcendent ‘bracketing’ of the external world as the phenomenological ‘epoché’ – a form of Cartesian doubt or action-disabling suspension of conviction (Beyer, 2018). Schutz’s epoché represents a mirroring complement to Husserl’s. In short, if the phenomenological epoché suspends a natural attitude involvement in the world via doubting the world, then the epoché of the natural attitude invokes an *impossibility* of doubting the world, evidenced via in-the-world action. In shared action, participants to the action jointly signal conviction in a shared, undoubtable world, a conviction more foundational than mere belief. It is this grounding investment in a common reality that provides conditions for common-sense coordination of and in a world-in-common.^5^

In the context of this ‘grounding reality’, Schutz described ‘multiple realities’, such as dream-worlds, abstractions of science, and worlds of myth and religion that all need to defer ultimately to the one ‘common-sense’ world of the natural attitude. Schutz called this paramount reality of the common-sense world ‘the world of working’ (1962b, 226), but we suggest that it is better understood as the mundane world of coordinated action – sitting in chairs, drinking from coffee cups, catching busses, and giving and receiving objects – the whole gamut of social actions, including those carried out in talk: delivering greetings, offering descriptions, making requests, etc (Schutz, 1962b; Psathas, 2014). Sass (2014a) has suggested that it is the loss of grounding status of this ‘paramount reality’ that creates the conditions for certain of the phenomena in *schizophrenia*. For instance, it is the loss of a grounding reality that in turn distorts the status of imaginary domains, so that the distinction between the imagined and the real is lost.

Certainly, other writers have discussed different worlds or realities: William James prior to Schutz and Goffman after him (Psathas, 2014); and within the philosophy of science, Popper (1978) and Penrose (2006) have independently developed three-world ontologies that bear some resemblance to certain aspects of our model. We, however, are making no ontological claims, and instead, in accord with Schutz, look to worlds as ‘meaning domains’ or global settings that social participants might orient toward in interaction. Nonetheless, while our model has its genesis in Schutz’s discussion of multiple realities, it ends up diverging considerably in the details.

### 2.1 A five-world model

Our model then ought to be read as a tool of interpretation – ‘worlds’ to mean ‘modes of world-revealing’, with each way-of-revealing sufficiently distinct as to suggest a different world-type. For those wary of introducing an interpretive frame, it ought to be pointed out that it is not so much introducing a frame as supplanting the one-world interpretive frame of common sense – ‘breaching’, in other words, the analyst’s own world-embeddedness of natural attitude investment in mundane reality.

We have given the worlds each a rather simple label to suggest that they lie within daily practices of languaging; their interweaving into a common-sense organization of the ‘one reality’ is a background, everyday affair, tacitly and socially accomplished. In everyday activity, we tend to jump seamlessly from one world to the next, mostly oblivious to the gulf we have just crossed. We describe them as ‘Me-world’, ‘This-world’, ‘That-world’, ‘The-world’, and ‘Beyond-realms’, and will gloss them now each in turn.

#### 2.1.1 Me-world

Me-world refers to the world of private, embodied experience. It is a simultaneous experiential coupling of a world-self relation within the enactivist understanding that ‘world’ and ‘self’ are co-arising phenomena, two faces of the one coin (Varela et al., 1991). It is not ‘my-world’, as the self does not stand constitutively before the experienced-world. If anything, self and world stand in an ‘as’ relation: self-as-world and world-as-self, both simultaneously constituted in experience, *as* experience. Husserlian phenomenology, as discussed, takes the self-world relation as its starting point for enquiry, and a particularly influential cluster of theoretical frames that posit ‘*schizophrenic* autism’ as a central feature or ‘generator’ of *schizophrenic* presentation makes this coupling of self and world (Me-world) of particular interest in phenomenological psychiatry (Minkowski, 1987; Parnas et al., 2002, 2005; Sass et al., 2017; De Haan and Gipps, 2018).

#### 2.1.2 This-world

Jakobson (2011), talks of indexical expressions as ‘shifters’, linguistic units whose meanings refuse definiteness of sense, ever retaining a context-dependent ‘pointing’ function. Garfinkel considered indexicality an ineluctable feature of social action and the core focus of ethnomethodological concern. The vast majority of sociological theory, in Garfinkel’s view, attempts to proceed by first ‘remedying’ or ‘fixing’ indexicality by substituting ‘objective for indexical expressions’ to ensure ‘rational accountability’ (Garfinkel and Sacks, 1970, p. 161). He called such approaches ‘remedial’ and contrasted them with the ethnomethodological intention to allow indexical ambiguity to remain as a key and dynamic feature of social organization.

Ineluctable indexicality is central to the discussion of ‘This-world’, which describes a world, as experienced, of immediate, indexical *haecceity*, or the irreducible specific ‘this-ness’ of the local situation in light of a fellow social member interacting with the same world. The local situation is a socially shared immediate context – a shared ‘immediacy-of-a-here’. It constitutes a reality insofar as Schutz’s epoché dictates that action presupposes solid ground, and it is a *shared* reality in that shared or mutual action demands that the ground of any such action must also be shared. The structure of that common ground however is perennially subject to immediate, microsocial negotiation. Joint attention is one of This-world’s key features; situational indexing is another. A successfully negotiated (social) reality of This-world involves members mutually orienting to commonly held formations of things, actions, and settings, which involves dynamic coordinations of ‘what-it-is-that-this-thing-is’ and ‘what-it-is-that-is-happening-here’. This is to say, members orient to the situated present of This-world via organizations of kind and Type^6^ that find form in language according to common understandings of relations between things, events, and settings. These collective understandings constitute social and cultural forms of background knowledge, variously called ‘common sense’, ‘mundane reason’, or ‘tacit knowledge’ (Pollner, 1987; Fuchs, 2001).

The ‘this-ness’ of an (experienced) object foregrounds its concrete and specific individuality above and in excess of its abstract category ascription; even if two people in a shared situation both encounter a creature as ‘a cat’, there is much in the creature that exceeds its category. The category, ‘cat’, is an abstraction; the creature before them both (*this* creature, the one that they can both point to) is concrete. ‘Selves’ are reconfigured as phenomena of intersubjectivity in This-world, and intersubjectivity – a primordial sociality – is explored in formal analyses through various methods of interaction analysis and microsociological microanalysis. If *schizophrenia* is to be conceived as a disturbance of common-sense (Blankenburg and Mishara, 2001) or a disturbance of intersubjective ‘between-ness’ (Phillips, 2001), then we should look for evidence of any such disturbed coordination in the immediate relations of This-world. Goffman (1983) describes the social dynamic of this shared immediacy as ‘The Interaction Order’.

#### 2.1.3 That-world

That-world is most easily discerned as an abstract background to This-world, a global domain of meaning which is non-immediate and non-present. Its defining mode of operation is as a socially coordinated and culturally shaped version of the real, but standing in contradistinction to the primordial sociality of the immediate present as well as the yet-to-be-discussed final two domains of reality. The attempt to ‘stabilize’ facticity, clear of subjectivity and indexical indeterminacy, is designed to establish ‘world-facts’ within social systems, or a ‘fact-world’ that need not be determinate (which is the key feature of ‘The-world’), but needs to be more stable than This-world.

Socially organized value systems and culturally determined hierarchies of relevance and preference play a role in this particular world-domain. If the prototypical operation of language in the mode of This-world negotiation is joint attention facilitated by indexing the immediate environment (e.g., pointing), then explicated symbolic formulations take over this role within That-world meaning-domains. With increased abstraction (which is to say, as we venture away from immediacies and further into the ‘That’), the indexical functions of language become text internal, pointing less to commonalities of experience and more to matters within the text itself, to culture, and common forms of life and knowledge. In an influential formulation, Goldstein (1959) described a key feature of *schizophrenia* as a disturbance in the *abstract attitude*, which might be considered, among other things, as discrete moments of disengagement from the concrete concerns of the immediate situation to re-formulate the frames of engagement. We might consider this a complex interplay between abstract and concrete world conceptions. Popper’s (1978) description of ‘world 3 objects’ in his tripartite ontology might also be said to belong to That-world.

#### 2.1.4 The-world

Set against Husserl’s conception of the life-world is a particular world conception that appears to eject the very nature of experience from its domain. Husserl attributed a ‘scientistic’ worldview as a natural consequence of a historical attitude he traced back to Galileo, who, he suggested, ‘formalize(d) nature by seeing it in terms of an abstract grid of mathematical quantities’, a point of departure that led to an ‘abstractive closure’ of the natural sciences ‘based on abstraction and formalization away from the concrete individual occurrences’ (Moran, 2012, p. 69). Such a world conception – i.e., an absolutist abstraction – shares qualities with aspects of described *schizophrenic* world-experience, namely de-animation (Stanghellini, 2004) and loss of dynamicism (Minkowski, 1987).

We can conceive of such a world as a determinate world against which our lived-worlds receive measure. Penrose (2006), in his three-world ontology, suggests that the world of mathematics represents an independent domain whose possibilities exceed the physical world and against only part of which the physical world maps. The deterministic domain of ‘The-world’ is a world of pure material determination. It constitutes a singular (universe-al) conception of states-of-affairs past, present, and future, held in a deterministic value-free chain of causation. It is world-as-object, the ground of the idea of observer-neutral ‘objectivity’, and it positions the human world and everyday affairs as being of the same fundamental type of determinate, de-animate phenomena. While The-world conceptions carry an aura of concreteness (they are about what ‘indisputably is’), they are actually highly abstract—the conceptual distance between the Big Bang and this-desk-here-before-me-as-I-use-it is categorical. The organization of my body as a concatenation of subatomic particles is inaccessible to my lived experience of the body as *this* body, the body that reaches reflexively for the keys of the computer keyboard, the hand that reaches up to scratch the head, the body that is hungry. The-world presents a cold, clockwork universe stripped of all possibilities of the fundamental feature of animacy. Within its modes of knowledge formation, the other domains of meaning are reduced to mere approximations of its own ways of world-revealing. By its very structure, The-world cannot allow itself to be conceived of as just one world among others.

#### 2.1.5 Beyond-realms

Beyond-realms are, as the term would suggest, set apart from the other four world domains, but with a distinct flavor of reality (they are ‘real’, but in a different way to the immanent domains). We shall not attempt to define them, except to say that they are referred to in language, and, as later analysis will detail, they need to be managed in language or by means of taboo and ritual to ensure that demarcation is maintained between the beyond and the mundane world. They share properties of transcendence with Me-world, and this has consequences in claims of mystic experience. We allow ‘Beyond’ as a placeholder for world-domains that might lie outside mundane world reference, in whatever way these might come up in interaction – and in *schizophrenic* interaction, they do so regularly. Rather than predefining them, we treat them as necessary backdrop for certain operations of language.

These five worlds have been delineated through close consideration of *schizophrenic* talk, and the suggestion was that we might account for certain features of *schizophrenic* interaction by positing a weakened sense of obligation to bring the different domains into coherent relation. Indexicality would seem to be the key organizing feature between the worlds, with it being possible to make the argument that the ‘view from nowhere’ implicit in ‘The-world’ removes the perspective-providing ‘I’ from the picture altogether.

## 3 Problems of naïve empiricism

In our attempt to identify meaningful patterns in the records of interaction, we encountered a series of problems. The most obvious was the danger of simply re-describing diagnostic parameters. *Schizophrenia* diagnosis is enacted, to a large extent, on the grounds of clinical interview, which is to say, upon factors evidenced in an interactional setting. A naïve approach to data analysis risks ‘discovering’ those very factors that were used to select the interview subjects in the first place. This presents an unhelpful circularity.

In addition, the heterogeneity of symptoms in *schizophrenia* presents a major obstacle when looking for patterns of interactional detail across different subjects or even within a single subject. In a monograph on *schizophrenic speech*, McKenna and Oh (2005) detail *schizophrenia* in terms of three semi-independent symptom clusters, or syndromes, delineated in terms of ‘positive’ and ‘negative’ symptoms, and a third factor, ‘disorganization’. One of the earlier intimations of this third syndrome referred to it not in terms of (a cognitive) ‘disorganization’ but rather as ‘disorders in relating’ (Strauss et al., 1974). This harks back to earlier descriptions by Jaspers and Schneider of a supposed difficulty in forming empathic bonds with *schizophrenia* diagnosees, possibly grounded in an ‘autistic’^7^ withdrawal from the field of relationships that Cameron (1938) claimed can lead ultimately to an encysted experiential bubble (Minkowski, 1987; De Haan and Gipps, 2018). Much language research in schizophrenia tends to focus on this third syndrome, conceptualized as cognitive disorganization or ‘Formal Thought Disorder’.

Nancy Andreasen redefined Thought Disorder as a ‘Language and Communication’ disorder via a scale of 18 descriptors (Andreasen, 1986). To repeat the earlier point: the danger of engaging in naïve interactional analysis without understanding what has already been operationalized as diagnostically significant is that of ‘unearthing’ exactly such diagnostic descriptors. An illustration might be drawn here with ‘clanging’, one of the 18 features on Andreasen’s scale that she describes as ‘A pattern of speech in which sounds rather than meaningful relationships appear to govern word choice... [involving] ...rhyming relationships... [and] ...punning associations’ (Andreasen, 1986, p. 478).

There are numerous examples of clanging in our data. One involves the responses of an interviewee, (BF), to a cognitive test run by the second interviewer (I2). The test proceeds by presenting the test subject with a non-sense word and offering a series of progressively more explicit clues, each time asking the interviewee to guess what the non-sense word means. BF is offered the test word ‘prither’, to which he responds with a confirmation/repair request 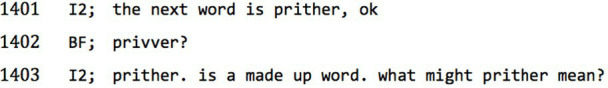
(1402):**data extract 1, Prither/enton1A**

Upon further clues being offered, the ‘game’ of question–response is continually derailed, and at one stage, BF asks to go for a cigarette break but under encouragement decides to continue with the final 
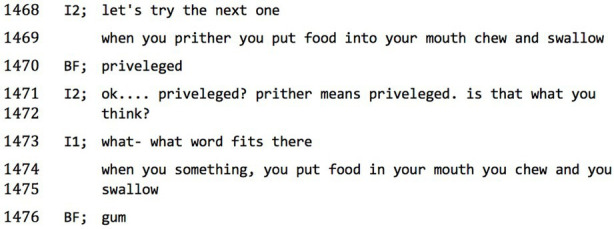
clue:**1B**

‘Prither’ (1401)⟶ ‘privver’ (1402) ⟶ ‘privileged’ (1470). The possible mishearing that triggered the repair initiation at 1402 continues to be the locus of orientation for BF, despite the intervening (but here elided) 50 lines of interaction.

To the following test word, ‘*enton*’, in combination with the meaning prompt of ‘*a form of art*’, BF responds
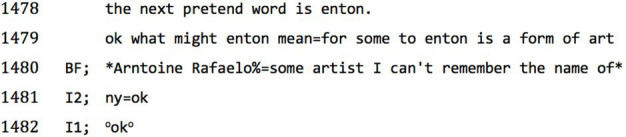
 ‘*Arntoine Rafaelo*’.**1C**

After this, he gets agitated, unclips the microphone and starts to talk into it in the manner of a sports commentator, describing what the interviewer is wearing. Asked whether he wants to have a break, he clips the microphone back on and asks to continue. The interview continues with the same test item, ‘*enton*’, and further clues elicit ‘*ten tonne hammer*’, ‘*newton*’, and ‘*per ten t’ tain*’, which the interviewer interprets as ‘*to entertain*’, and finally a string of music-themed and alliterative words and sonic fragments – ‘*melody, melodic(s) tones ‘n’ moes*’.

It is obvious that in these responses, BF is responding more to phonological cues than to semantic associations. But to let the observation rest here would be to simply redescribe Andreasen’s diagnostically significant ‘clanging’. On the other hand, seen in the context of a progressive deterioration of the ‘interview game’ and BF’s attempts to leave the interview, we might also interpret this gathering swarm of phonological associations as performing work of resisting, avoiding, or otherwise displacing semantic coordination. Which is to say, it is not in itself meaningless.

Harvey Sacks is generally credited as the founder of Conversation Analysis. In an intriguing article based on a talk presented shortly after he died in 1975, Gail Jefferson details ‘exploratory’ work on poetics that Sacks had been engaged in in the final years of his career. She describes motivation for the talk thus:

...the field of Conversation Analysis was coming to be identified almost exclusively by reference to the Sacks et al. paper "A simplest systematics for the organization of turn-taking for conversation" published in 1974. As an antidote to that drastically constricted version of the field, I decided to present the wild side... (Jefferson, 1996, p. 2)

She introduces the tentative work on poetics with a consideration of psychotic talk but goes on to detail similar phenomena appearing in ‘ordinary’ talk. She later notes that there is something ‘autistic’ about the self-referential nature of some of the sound and categorial associations discussed, in both psychotic and normal talk. However, she cites an early psychiatric researcher to claim that what sets psychotic production apart from ‘normal’ – what constitutes the pathology, in other words – is not so much the formal feature of textual self-reference as ‘the tendency to incorporate such autistic productions without any endeavor to translate them into a form which considers the needs of the listener’ (Woods, 1938, p. 302). In Conversation Analytic terms, this attention to the needs of the listener is described as ‘recipient design’ (Sacks et al., 1974).

What follows in Jefferson’s article is a series of descriptions of poetic instances that stand outside the normal constraints of Conversation Analytic methods: observations, hunches, and interpretations that verge on psychological readings. Jefferson is unapologetic, quoting Sacks’ response to criticism that such noticings might get ‘carried too far’ by noting that, first, one needs to raise them as a possibility. The work was exploratory, Jefferson stressed, and so one needed to ‘push the stuff, keep pushing at it, see how far it might go, you can always pull back to a more cautious, reasonable, sensible position’ (1996, p. 9).

If we look back at the above series of data extracts as a loss of recipient design, a collapse of the relational field into autistic textual self-reference, then we might ask – what use are Conversation Analytical tools here? Jefferson provides a tentative answer: We are exploring the boundaries of the Conversation Analytic method. Suggesting that there might be another world of significance intruding here – an autistic ‘Me-world’ – is to peer over the fence-line of Conversation Analytic (‘This-world’) concern and see what might be pushing back against the fence from the other side.

## 4 Three methodological hurdles

In a classic article on aphasia, Jakobson (1956) argued that the study of language breakdown in pathology might lead to better understanding of normal language function. However, linguistic interest in the language anomalies of *schizophrenia* has remained slight. In addition, what studies have been carried out have tended to focus on elicited forms of decontextualized production and clinically set forms of talk rather than on natural language interaction. In performing Conversation Analysis on topic management in unstructured talk between diagnosees and close relatives, Riou (2015) was able to demonstrate that the interactive dyad could employ non-canonical strategies to progress a conversation despite ‘glitches’ in topic transition and suggests that a richer approach to *schizophrenic* talk (and atypical interaction more generally) might involve identifying such idiosyncrasies of interaction as an adjunct to descriptions of dysfunctions of language production. This focus on the interactive dyad rather than the features of the abstracted language of an atomized psyche offers both research and therapeutic potential. McCabe et al. (2004) and McCabe (2009), for example, were able to argue against an influential ‘Theory of Mind’ account for *schizophrenia* (Frith and Corcoran, 1996) by applying Conversation Analysis to transcripts of diagnosee interaction.

It should be noted, however, that applying the Conversation Analytic method to *schizophrenic* interaction is not without its problems. The first, as discussed in detail by Riou (2015), is the difficulty in accessing conversational data on account of the vulnerable population. A specification of this difficulty is in accessing data that involves interactions with ‘normal’, non-clinician interlocutors. The data used in the current study falls somewhat short of this ideal of ‘natural’ conversation. As mentioned, the recorded interactions did not involve clinical settings of examination, diagnosis, or treatment but were introduced to subjects as being for the sake of non-specified ‘language research’. Nonetheless, the primary interviewer was a clinical psychiatrist member of the mental health service from which subjects were drawn, and interviews were semi-scripted, employing a combination of open-ended question prompts as recommended by Andreasen (1986) for eliciting language production for diagnostic purposes, as well as a series of more formalized cognitive test procedures introduced by the secondary interviewer. The cognitive framing of the researchers’ motivating interests led to efforts to stimulate monologues or extended turns in an attempt to minimize interviewer ‘intrusion’ into the data, which is not ideal from the perspective of interaction analysis.

In addition to these general concerns, we identified three more specific ‘hurdles’ of method that needed to be addressed.

### 4.1 Hurdle 1: weakening of the next turn proof procedure

Schutz (1962a) distinguishes social sciences from the natural sciences by seeing the former as involving the study of human meaning-activity whereas objects of interest for the physical sciences are inanimate. In contrast, objects of social scientific interest involve future-directedness and organizations of relevance and meaning. He describes these meaning-organizations of social scientific interest (the ‘objects’ of social science) as ‘first order’ meanings. Because they are grounded in biological activity, these objects of the social sciences are inherently organism-orienting, relevantising, and interpretive. Schutz calls the products of any such enquiry – which is to say, the meanings of a social scientific discourse – as ‘second-order’ meanings (meaning of meanings). As meaning-activities, these second-order meanings are likewise grounded in biological activity, and so, are likewise inherently organism-orienting, relevantising, and interpretive.

CA method attempts to access first-order meanings of the interactional situation directly, without imposing second-order meanings upon the phenomena through projection of macro-theoretical categories in a manner that Schegloff (1997, p. 167) critiques as ‘theoretical imperialism’. It has primarily done this by focusing analytical attention (and, therefore, interpretive machinery) not on the lone utterance but instead by looking for evidence of meaning orientations and displays of understanding in interlocutor responses. The ‘next turn proof procedure’ recommends understanding the interactional meaning of a particular utterance by looking to see how an actual participant in the interaction interprets it, as shown through the manner in which they formulate their following turn. As Hutchby and Wooffitt (2008, p. 14) state, ‘any ‘next’ turn in a sequence displays its producer’s understanding of the ‘prior’ turn, and if that understanding happens to be incorrect, that in itself can be displayed in the following turn in the sequence’. Nick Enfield has pointed out that the ‘proof procedure’ is actually a ‘disproof procedure’, given the opportunity in the third turn to correct a misunderstanding (pc cited in Levinson, 2012, p. 129). This offers an elegant statement of social interaction as a ‘mundane’ form of social science: coordinated meanings are never settled or positively proven, but always contingent, existing in a dynamic state of provisional acceptance and ongoing negotiation, just as formal scientific hypotheses are.

The “next turn proof procedure” was first described by Sacks, Schegloff, and Jefferson–

… while understandings of other turns’ talk are displayed to coparticipants, they are available as well to professional analysts who are thereby afforded a proof criterion (and a search procedure) for the analysis of what a turn’s talk is occupied with... it is the parties’ understandings of prior turns’ talk... that are wanted for analysis. [this] affords…a proof procedure for professional analysis... (Sacks et al., 1974, pp. 728–729).

Of course, the reading of interlocutor orientations to ‘what a turn’s talk is occupied with’ involves analyst interpretation, but it is an interpretation held in check by reference to the *following* participant turn, and so on, within the ongoing progressivity of talk and within the immediate context of relevance that is itself constantly being created and managed by participants-to-the-interaction within the interactional setting, in which the analyst plays no part.

A corollary of next turn evidencing is that speakers design their turns to fit the preceding turn. This would appear to suggest a ‘rule’ that, to assure coherence, next turns are to an extent determined by that which preceded them in concert with situational context. In practice, however, ‘next turn’ productions are potentially infinite – what provides the guard rails of constraint are situationally specific expectations of accountability within an ongoing collaborative project of context construction and management that is indigenous to the interaction itself (Stokoe et al., 2021). One can say *anything* on a following turn – but if it diverges too far from expectations, one will be held to account for it.

While CA seeks to privilege in its analysis ‘the orientations, meanings, interpretations, understandings etc. of the participants’ (Schegloff, 1997, p. 166), making analytic judgments as to what participants display as their understanding of a previous turn itself relies on the implicit assumption that analysts share enough of the language and cultural background of the participants to confidently assign meaning to turns. Ethnomethodology makes explicit use of this in its recommendation of ‘self-reflection’ as a tool to recruit the analyst’s own expertise in common-sense understanding as an interpretive resource – specifically, in drawing out webs of assumed-and-taken-for-granted implicature (Francis and Hester, 2004) – however, within the strictures of CA method, this ‘mundane expertise’ of the analyst remains implicit.

The notion of “normativity” comes into play here: the fact that the analyst is an ‘everyday expert’ of common-sense organization of talk – the very same expertise that allows participants of everyday talk to order and coordinate their first-order meanings – means that the analyst is equally, as a matter of mundane expertise, able to recognize what is ‘non-normative’.

#### 4.1.1 Non-normativity

In the case of delusional discourse in *schizophrenia*, Palmer (2000) has made the point that judgments of psychiatric pathology require an understanding of appropriate context-dependent social norms and that patient actions will be seen to take on symptomatic significance when they are judged as contravening these social norms. Palmer leverages studies by Wooffitt (1992) on ‘normative’ tellings of ‘paranormal’ events (experiences of ghosts and such – what we might call ‘Beyond’ phenomena) to show that it is not so much the content of certain delusional accounts that marks them as pathological as it is their non-normative management in the telling. But the warrant to judge such normative transgression is not limited to psychiatric specialists, and it is a matter of mundane expertise held by all reasonable practitioners of common sense. Smith (1978) describes how friends, family, and associates of a social member (‘K’) funnel K toward psychiatric services on the basis of pre-diagnostic attributions of acting ‘queer’, being ‘impractical’, ‘out of touch’, and having ‘foibles’, with the normativity transgression implied by these judgments finally made explicit when her behavior is ultimately described as ‘not as it should be’ (Smith, 1978, p. 31). Such non-diagnostic recognitions of the non-normativity of certain aspects of talk-in-interaction with *schizophrenia* diagnosees we here refer to as a recognizable ‘madness’. Its recognition is a matter of everyday skill for social practitioners whose interactive practices are predicated upon the very normativity that the madness is seen to be in breach of. We make a distinction then between the social organizations of *schizophrenia* that flow from a diagnostic speech act and the non-formalized social organizations of madness.

Considering talk-in-interaction in terms of normativity directs our attention toward various types of non-normative interactions in various atypical populations. Antaki and Wilkinson, in their overview of CA and interactions involving atypical populations (Antaki and Wilkinson, 2012), specifically state that Conversation Analysis might not be able to say as much about atypical interaction as about ‘typical’ interactions. One reason for this may be, as was found in the current study, a weakening of the next turn proof procedure. Antaki and Wilkinson note that in a study of the interaction between psychiatrists and *schizophrenia* diagnosees by McCabe et al. (2002), ‘one pervasive feature... is the doctor’s markedly neutral reception of the client’s news announcements, when these are hearably ‘*mad*”(Antaki and Wilkinson, 2012, p. 545). The ‘hearable *mad*ness’ that Antaki and Wilkinson refer to represents, of course, the imposition of a category ascription by an overhearing analyst. The fact that the analyst orients to normativity’s breaching here, but that the clinical interlocutor does not orient to the same breaching, presents something of a problem. The doctor’s *lack* of response stands in stark contrast to the findings of Garfinkel’s well-known breaching studies (Garfinkel, 1967), where deliberate but relatively minor ‘breaches’ of normativity by experimental stooges drew extreme reactions from interlocutors, leading Garfinkel to claim that the normativity of the interaction order represents a *moral* order. From this, we might surmise that at a certain ‘tipping point’ of escalating ‘madness’, the mechanisms of social accountability start to break down, and interlocutors fail to be held to account, however subtly, for transgressions of social order when they start to be oriented-to as mentally ill. In this failure of accountability, we start to see a weakening of the basis for the next turn proof procedure.

We might see evidence for this weakening of next turn proof in the following extract from our 
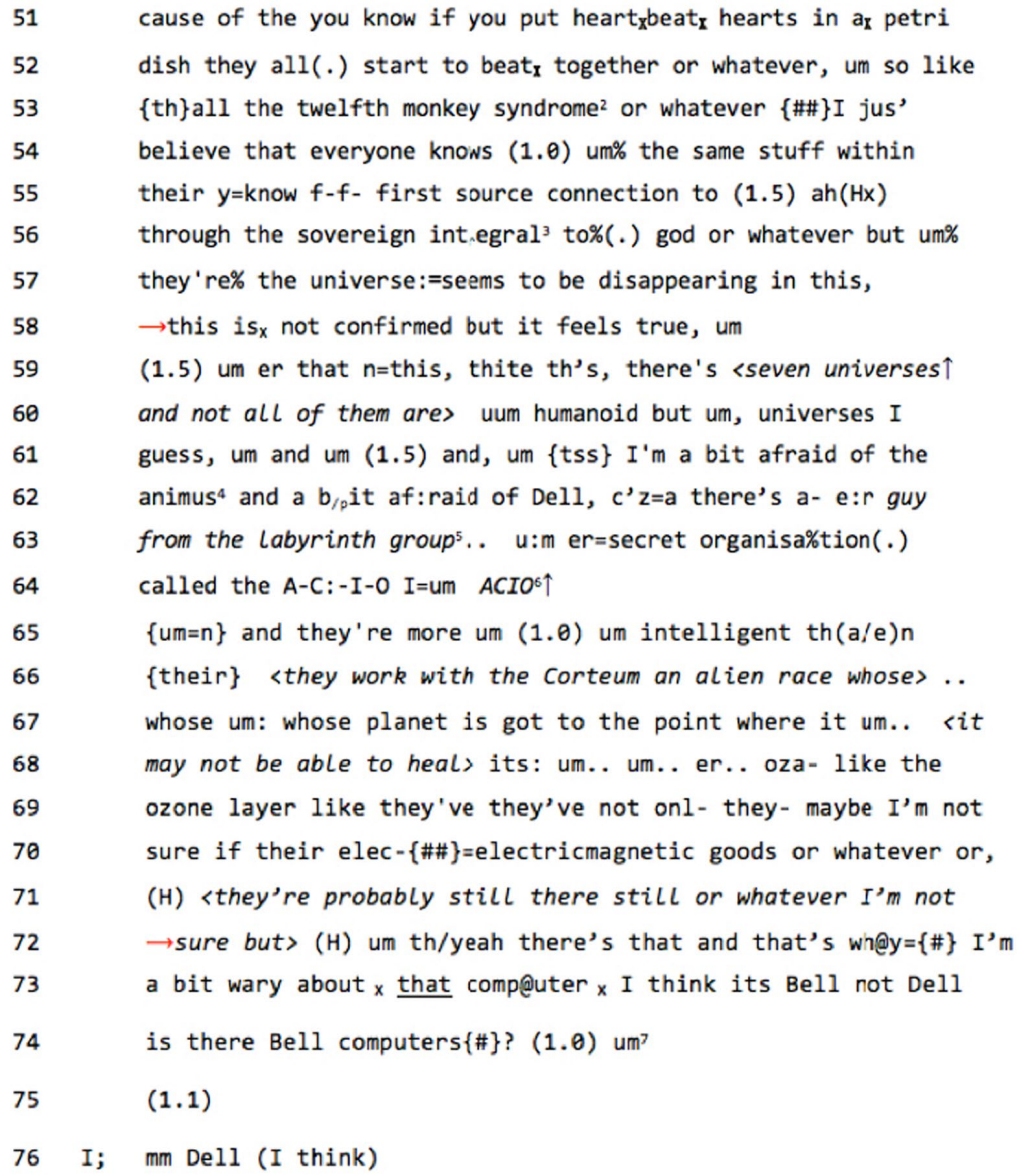
data base:**data extract 2, ‘seven universes’**

The interviewer (I) in this extract is noticeable in their lack of co-constructive input. There is no verbal input for 23 lines (we lack information about the non-verbal) until they are asked a direct question by the interviewee – and even then, there is a 1.0-s gap at line 74 followed by a 1.1-s gap at 75 before the interviewer responds at 76 with an epistemically hedged reference to the most mundane aspect of the interviewee’s account, with no orientation toward the bizarre metaphysics – the ‘recognizable madness’ – that has gone before.

This leaves us little to fall back on in terms of next turn evidencing. We might, however, look to within-turn features for evidence of recipient design (Drew, 2013). In the previously mentioned studies by Wooffitt (1992), it was noted that in telling of ‘paranormal’ experience, narrators need to establish their credentials as credible members of a common sense community before embarking on their telling of things and happenings that lie beyond the bounds of the mundane world. There is an interactive need, in other words, in the telling of paranormal experience, for the teller to establish themselves as hearably ‘not-mad’, before embarking on topics that might possibly be perceived as mad, and to bracket out the paranormal (the ‘Beyond’) from the normal and mundane. Wooffitt identified a mundanity marking structure – ‘*I was just doing X*’ – that provides mundane context for the paranormal event, ‘Y’, so that the formal structure of such a telling might be seen as: ‘*I was just doing X, when Y*’. In other words, there is a prior grounding in common-sense, as well as a ‘ritualized’ demarcation of the paranormal that is necessary to maintain the recognizable mundanity of the mundane world. Palmer (2000) pointed out that it was just such sectioning off of the paranormal from the normal that was lacking in one analyzed extract of diagnostically delusional talk, where the interviewee describes having met a god, who ‘calls himself Thor’, and Thor is introduced to the telling with much the same matter-of-fact manner as the good, socially accounted, common-sense character not a few lines later of ‘Mr Burnett the animal food manufacturer’.

If we return to data extract 2, we find that some of the ‘metaphysical’ content between lines 59 and 72 has been bookended by two indexical constructions – ‘*this is not confirmed but it feels true*’ (58) and ‘*there’s that and that’s why I’m a bit wary about that computer*’ (72). The material between these two markers might be considered ‘hearably mad’, so the speaker might be considered to be performing interactive work attempting to manage this with the indexical marking. This reading receives support when his use of the mundanity marker ‘*jus”* in line 53 is taken into account, as well as the speaker’s efforts to ground his belief in common sense ‘*everyone knows (1.0) um% the same stuff* ’, and the epistemic hedging that leads up to the supra-normal account via dysfluencies of pausing, fillers, and false starts (52–57), as well as explicit marking of uncertainty with ‘*seems*’ (57) and ‘*this is not confirmed but it feels true*’. This epistemic hedging continues with increasing frequency toward the end of the account between lines 68 and 72. In effect, JX can be seen to be going to great lengths to manage the intersubjective contentiousness of his candidate cosmology, and he directs his audience toward those specific epistemic domains that he believes provide evidence for the account: the domain of direct experience where ‘*it feels true*’ and the domain available to everyone of ‘*first source connection*’ where ‘*everyone knows the same stuff*’. Thus, he is attempting to bolster his metaphysical claims by leveraging them away from the ‘merely subjective’ to their being epistemically grounded in common sense in an attempt to intersubjectively stabilize the claims, giving them sway over the mutually-revealed interactionally-relevant world of the shared situation.

The effect of all this work is that the speaker appears to be anticipating interlocutor disagreement and pre-emptively managing it, displaying in the process a delicate level of attunement to the interactive space, despite the ‘hearably mad’ content. There is, however, a similar co-mingling of the ‘Beyond’ with the mundane, as Palmer noted: stories of the ‘Corteum’ alien race mingle with mentions of electromagnetic goods and ‘Bell’ computers with no noticeable shift of story-world or setting. There are resonances here with phenomena that have long been recognized in the psychiatric literature and described in terms of ‘double bookkeeping’ (Sass, 2014a), where diagnosees appear to maintain two different world accounts concurrently, such that, for instance, a hospital patient who might claim to be the Queen of England will nonetheless line up patiently for dinner with other patients – one foot in the ‘delusional’ world and the other in a world of shared immediacies.

The argument presented to this point has been that with the loss of the mechanics of accountability for breaches in normativity, the next turn proof procedure is noticeably weakened. This raises the question of how a Conversation Analytic approach to interaction analysis might proceed when its most useful tool has been blunted. The above analysis has used features of turn design (Drew, 2013) to analyze the interactive orientations of an extended turn, but in addition to this, it has also seen the need to enlist analyst sensitivity to breaches of normativity to identify the ‘hearably mad’ in the absence of interlocutor responses. It has also leaned on the psychiatric attribution of ‘delusion’ and referred to constructs within psychiatric literature (double book-keeping) to suggest a sense-making frame. Such moves align with Garfinkel’s ‘unique adequacy’ requirement—which stipulates that in order to study specific domains of praxis, it behooves the ethnomethodological analyst to have at least some minimal experience of the domain, of its practices, its language, and its organizational structures. In adopting such tactical responses, the analysis moves away from Conversation Analytic methods toward what Pollner (1974, 1975) describes as an ‘ethnomethodological attitude’ and also toward dialectic engagement with psychiatric discourses.

### 4.2 Hurdle 2: unexamined importation of social categories (de-reifying the construct)

It is of central importance that while the analyst, as everyday ‘expert’ in common sense, might orient to ongoing breaches of normativity as ‘hearable madness’, it is most often the case that the psychotic speaker themselves will not. This warrants examination in terms of a frame conflict that appears to lie at the heart of certain *schizophrenic* phenomena and is generally referred to as a lack of ‘insight’.

Subject selection for the ‘analysis’ considered here had already been performed, self-evidently, on the basis of psychiatric diagnosis. This preselection represents a social organization – a delineation of person-Type that carries implicitly a background social theory of failed membership (Smith, 1978) as well as implicit attributions of pathological meaning-organization (Von Bertalanffy, 1960). These attributions and pathologies have been ascribed from the outset via the diagnostic speech act to the individual diagnosee as an isolated entity, a dysfunctioning *psyche*. This psychological framing masks the social ordering implicit in the institutionally mandated speech acts of diagnosis and construct delineation. If left unexamined, this ordering of the social field is imported into the analysis at the very outset.

The change in perspective required here is revealed by considering a shift that occurred in the working definitions of ‘schizophrenia’ during the reported study’s development. What was initially conceived, unproblematically, in terms of symptom descriptors, where *schizophrenia* would be described in terms of ‘delusion’, ‘hallucination’, and ‘disordered thinking’ (at various levels of descriptive detail), came to be reconceptualized as a complex process of category ascription that involved a funneling of various phenomena of social breaching toward mundane ascriptions of social liminality (‘madness’) and ultimately toward institutionally mandated speech acts of formal diagnosis. To put it very simply, in place of being conceptualized as a list of symptoms, *schizophrenia* came to be seen as something that one socially organized person-Type (psychiatrist-Type-members) does to another socially organized person-Type (*schizophrenia*-Type-members).

It should be noted that such a perspective aligns rather closely with the perspective of diagnosees who are described diagnostically as ‘lacking insight’, which is to say, who disagree with their diagnosis. While ‘lack of insight’ is not monolithic and varies both in intensity and form, it is generally attributed to between 50 and 80% of people who receive a diagnosis of *schizophrenia* (Amador and David, 1998). ‘Lack of insight’ represents a site of frame conflict between institutional psychiatry and diagnosees: the person who might be on the receiving end of an unwanted (and from their perspective unwarranted) diagnosis indeed can perceive ‘schizophrenia’ as (nothing more than) a social categorization that is performed upon them by institutionally mandated others. Problems in meaning coordination in *schizophrenic* interaction will appear from the clinical perspective as failures in meaning production on behalf of the patient, but from the patient’s perspective, these can present as failures of meaning reception on behalf of the clinician (Rochester and Martin, 1979). The interaction analyst who approaches interactional data unreflectively risks importing the psychiatric stance – the ‘psychiatric gaze’ – at the very ground of the project. Jeff Coulter (1973, 1991) discusses this in terms of a reification of the *schizophrenia* construct, and his recommendations for avoiding it include the analyst engaging in a Wittgensteinian type of ‘conceptual clarification’. Attempting to address this problem at its root means taking into consideration the diagnosee’s ‘lived experience’, and in the current project, this has involved a qualified exploration of phenomenological methods, in particular, clarifying what role Husserl’s notion of the life-world might play in generating understandings of the interactional data.

### 4.3 Hurdle 3: phenomenological (life-world) considerations

Hepburn and Potter, in outlining Conversation Analysis as a qualitative method within psychological research, suggest that within the situational specifics of institutional settings, ‘it is important to seek insights into the participants and their roles’ (Hepburn and Potter, 2021, p. 18). They also note – an important addendum within the psychological context – ‘the focus [in CA] is on settings rather than people’ (2021, p. 15).

Consider then the following first-person description (translated from the original French) of schizophrenic experience from a well-known published account of a pseudonymous ‘Renee’:

For me, madness was definitely not a condition or illness; I did not believe I was ill. It was rather a country, opposed to Reality, where reigned an implacable light, blinding, leaving no place for shadow; an immense space without boundary, limitless, flat; a mineral, lunar country, cold as the wastes of the North Pole. In this stretching emptiness, all is unchangeable, immobile, congealed, crystallized. Objects are stage trappings, placed here and there, geometric cubes without meaning.

People turn weirdly about, they make gestures, movements without sense; they are phantoms whirling on an infinite plain, crushed by the pitiless electric light. And I—I am lost in it, isolated, cold, stripped purposeless under the light (Sechehaye, 1968, p. 44).

Renee explicitly addresses the institutional frame of psychiatry: ‘madness’ for her is not an *illness*. This *for-her* aspect – a ‘lived experience’ of *schizophrenia* – nudges us toward phenomenological considerations. Within phenomenological psychiatry, *schizophrenia* is often framed as self-disturbance (technically, an ‘ipseity’-disturbance, see Sass and Parnas, 2001; Sass, 2014b), but Renee does not describe it in this way. She describes her experience of madness instead as a ‘country’, inhabited by meaning-depleted ‘stage trappings’. She orients to madness, within this description, not in terms of personhood, but in terms of an alteration of settingedness. What is under description here is not a delusion, a hallucination, or even a disturbed sense of self, but rather a particular type of *world-*experience.

In Jefferson’s above-mentioned ‘wild’ foray into poetics, she relegates to an appendix her ‘wildest’ observation, ‘so improbable that presenting it [might] simply impeach anything else I might say’ (Jefferson, 1996, p. 49). It concerns two separate attempts in different interactive contexts by the same person, ‘Emma’ (who, it needs to be noted, is not psychotic), to index a personally significant setting in conversation. In both cases, the conversation circles around television coverage of the assassination of Robert Kennedy when, with little preparatory work, Emma announces that ‘that’ – meaning the spot where Robert Kennedy’s body was loaded onto a plane – was the same spot from which she, Emma, had taken off on a plane for a 
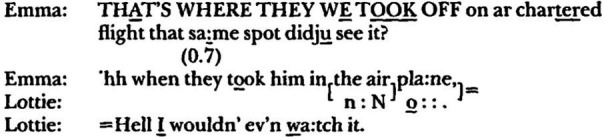
trip 
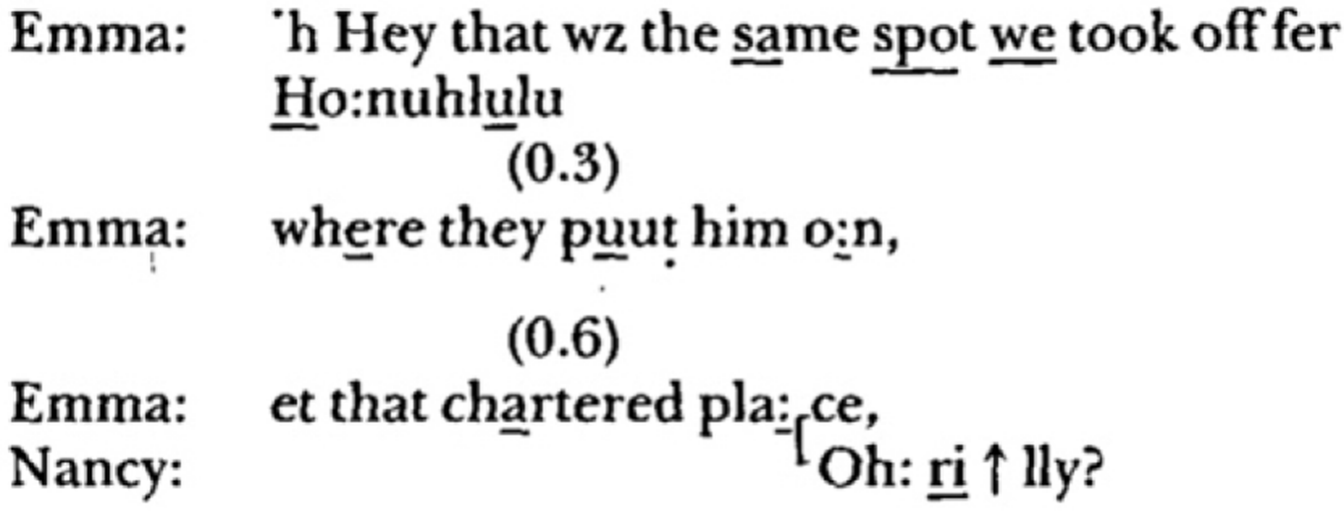
to Honolulu.**data extract 3, ‘internal landscape #1’, from Jefferson (1996, 53)** **data extract 4, ‘internal landscape #2’, from Jefferson (1996, 53)**

Jefferson makes the following observations–

Each of the announcements is formed up in the same way. Emma is pointing at something, "that spot," as if she and her recipient were passengers on a bus, and she's noticing a feature of the landscape. And in each case her recipient has difficulty locating what's being pointed to... It may be that Emma is indeed pointing to a feature of the landscape, but a landscape accessible only to her; an internal landscape. And it may be that the feature of the internal landscape that she's pointing to is present in the words that immediately precede each announcement (Jefferson, 1996, pp. 53–54).

Jefferson then searches for cues in the prior text that might have acted as triggers to place Emma in that landscape but which failed to similarly place her interlocutors, something to account for the ‘enigmatic pointing to something that just is not there’. At this point, the interlocutors are in two worlds, the world of historical significance (That-world in which Kennedy is assassinated) and the (Me-)world of personal significance, and Emma is attempting to bring them together, to place herself within the historical context and establish her ‘brush with history’ (Jefferson, 1996, p. 56). Note within this context the ‘THEY ⟶WE’ pronoun repair in the first line of the first example. That she is unsuccessful in bringing her interlocutor along with her in the first example is sufficient for Jefferson to point out the parallels of this ‘enigmatic pointing’ with the autism of psychotic talk, which proceeds ‘without any endeavor to translate [it] into a form which considers the needs of a listener’ (Woods, 1938, p. 302).^8^

Wouter Kusters, a Danish linguist and philosopher who has experienced two episodes of psychosis describes as a metaphor for his experience of psychosis a machine from a science fiction novel – a ‘Rhennius machine’ – which transforms objects into their mirror image. A left shoe, if placed in the machine, returns as a right shoe (Kusters, 2020). If a person steps into the machine, they, similarly, come out with everything flipped right-to-left, even modes of perception and ways of thinking become ‘flipped’, and this leads to the interesting point: to a person thus mirror-inverted, it is the whole world that appears to have undergone a transformation – cars drive on the opposite side of the road, doors open contrariwise, etc. Their ‘Me-world’ has undergone a paradigm shift, while ours will have remained as they were.

The point of Kusters’ metaphor is that we can see the situation of the mirror-inverted perceiver by way of two different aspects. One, which presents itself as objective, is the view from the non-mirror world, where we should say that the person has been reversed; the other is from the perspective of the inverted perceiver themselves, who can reasonably claim to have remained the same while the entire world has undergone a mirror inversion. We, as non-participant observers of this fictional world, can perform an ‘aspect-switch’ from seeing the scenario in one way via a complete and instantaneous reorganization, to seeing it via an alternative aspect. By means of the device, Kusters aims to undermine positivist conceptions of psychosis, which would approach presentations of psychotic phenomena from an ontologically stable frame of reference in the realist tradition. Such an approach fails to take into account the meaning world (the life-world) of the mirror-flipped person. Interaction with a psychotic patient then might be likened to trying to talk to a ‘Rhennius machine traveler’, while the traveler in talking to us is attempting to make sense with someone who, from their perspective, is in a reversed world. A common language fails because the assumption of grounding reference in a common world has failed.

The thought experiment takes to the extreme the same phenomena of ‘internal landscapes’ that Jefferson was exploring in the above examples. Paying attention to the possibility of these internal landscapes, we claim, means paying at least some analytical attention to the existence of an experiential life-world of diagnosees, and the work that goes into, or fails to go into, integrating this experiential life-world into a life-world that is shared (and sensed) in common with their interlocutor(s).

What, as analysts of talk-in-interaction, might we take from these considerations in approaching the study of interaction in *schizophrenia*?

Certainly, it is not our task to try to ‘get inside’ the experience of such a ‘world-flipped’ person. As Anderson et al. point out, ‘capturing and expressing the nature of the individual’s experience is not ethnomethodology’s topic’ (Anderson et al., 1985, p. 244). But it does appear to task us with examining our own ontological assumptions to stop us from projecting them onto the other’s experience and meaning formations. Similarly, our task is not to talk to such a person across the difference in world construal (this might be considered a task of clinical psychiatry) but instead to examine how talk proceeds between people who might inhabit different worlds without taking out *a priori* investments in the ontological grounds of either world. Thus, part of our task must involve an investigation of our own world assumptions. This is the task that Coulter identified as ‘conceptual clarification’, and which Pollner (1974, 1975) has pointed out involves a necessary distancing from common sense and the ‘undoubtable’ single world of its paramount reality. This is work that the manifold-world model has been proposed to perform. As Garfinkel demonstrated, common sense needs to be ‘breached’ before it can be seen. This is bound to be unsettling.

## 5 Proceeding on the basis of a gloss

Abiding by Garfinkel and Sacks’ recommendations on ‘glossing procedures’(Garfinkel and Sacks, 1970, pp. 164–165), we are loath to fix by definition the ‘worlds’ that we have sketched in outline; instead, we would look for examples of occasioned use to unpack their implications. It ought to be clear by this point that This-world is the domain within which microsocial interaction analysis plays. Perhaps less clear is that That-world includes macrosocial and institutional forms of organization. In this section, we will focus on explicating relations and translations between these two worlds.

Eglin (2017) does some of this work for us when he describes Garfinkel’s studies into the work of the Los Angeles Suicide Prevention Center, where investigators had to establish an account of death, as dealing with the ways that details of the ‘thises’ were processed into a formally accepted account of death:

‘A “that” – the social fact of a suicide, for example – is made up of a bunch of thises. The relationship among the thises and the that is not correlational or causal but...involv(es) the mutual determination of meaning as in the documentary method of interpretation’ (Eglin, 2017, p. 8).

The documentary method of interpretation, originally attributed to Mannheim (Roth, 2015), consists of treating an actual appearance or phenomenon as a ‘document’ or instantiation of an underlying regularity or pattern.

...the coroner...must make their determinations ‘with respect to the ‘thises’: they have to start with *this* much; *this* sight; *this* note; *this* collection of whatever is at hand (Garfinkel, 1974, p18, as cited in Eglin, 2017).

The interplay between details of the ‘this’ and socially consolidated details of the ‘that’, as Eglin describes, is a circular method of mutual interpretation between ‘this’ of instance and ‘that’ of underlying pattern–

...history itself...has been a bunch of thises and that's. Ethnomethodological studies...[are] irremediably tied to the ‘thises’ insofar as, through members’ methods of sociological enquiry, they ceaselessly transform into “thats,” and to the “thats” that give “thises” their meaning (Eglin, 2017, p. 26).

*“‘Thats’’ that give ‘thises’ their meaning*” might be understood here to mean that the underlying abstract patterns (‘thats’) toward which actual concrete events (‘thises’) point represent Typifications, so that the specificity of any actual event might be characterized in terms of patterns of ‘*what-it-is-that-is-happening-here’*, and its situated elements in terms of ‘*what-it-is-that-this-thing-is*’ – which is to say, in terms of a *Type* of happening or thing.

It bears repeating that formulation of the ‘five-world’ framework emerged from considering language use of people who had received a diagnosis of *schizophrenia* – which is to say, it was a pattern discerned from a handful of ‘thises’. Aspects of *schizophrenic* talk, it was suggested, involved a loss of integration between these five domains of meaning and a subsequent loss of common sense world-organization. The following ‘analyses’ of interactive data are presented as illustrative of this suggestion.

### 5.1 What was that knock?

We present these next two extracts between a diagnosee (BF) and the chief interviewer (I1) as examples of similar phenomena, negotiation over elements of the situated setting. The first might be considered a successful negotiation, resulting in participants being able to ‘go on’ with the business at hand (see Sterponi and Fasulo, 2010), and the second escalated into interactional trouble. In the first, we suggest that the interaction was progressed despite BF displaying an apparent ‘role reversal’ – reading the situation via a ‘That-world’ schema that appears contrary to common-sense, but which the interviewer does not orient to as in any way unexpected, actually acceding to the role reversal in terms of epistemic organization. In the second, there is a dispute over the ‘That-world’ nature of a recording device. Here, the interviewer stands his epistemic ground *as* an interviewer, insisting that the device is to record the conversation and not medical information. BF explicitly states his disagreement in an escalation of conversational 
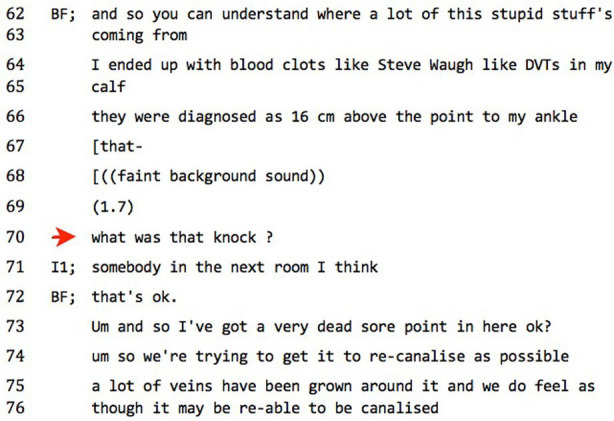
trouble.**data extract 5, ‘what was that knock?’**

BF has been claiming that he is on the ward because of a sports injury that he has been treating with traditional Chinese medicine and Shiatsu massage – an alternative frame, we can safely assume, to the medical account for his psychiatric hospitalization. Between lines 64 and 66, he is orienting to his body as a medicalized (The-world) object with a total lack of epistemic hedging that contrasts the interviewer’s hedge (‘*I think*’) in line 71. He appears to adopt a stance of reversed institutional role, with pedagogical comprehension checks at 62 (‘*you can understand’*) and 73 (‘*ok?*’), and is in the process of providing an account of his self-diagnosis when there is a sound at 68. BF interrupts his account and orients to the sound, asking at 70 after a long pause (1.7 s) what it was. The interviewer suggests a candidate source: ‘*somebody in the next room I think*’. BF’s response at 72, ‘*that’s ok*’, rings a little odd to us, although the interviewer did not respond to it as odd. BF then returns to the account of his claimed injuries and self-treatment, self-selecting at line 73 with an indexically signposted (‘*here’*) Me-world body-account — over which he has sole epistemic authority – in first-person singular (‘*I*’), that then transitions back to a medicalized (The-world) account with an accompanying shift to first-person plural pronoun (‘*we*’).

When BF orients to the strange noise, the interviewer provides an epistemically hedged account, and BF accepts the account (although, as suggested, in a ‘hearably odd’ manner) and continues with his prior activity, although with a shift in world-domain to Me-world. The mystery element has been integrated into the shared situation in a mutual enough manner in order for the participants to be able to go on with the activity. This is an example of This-world negotiation. Contextual phenomena, which would normally be the background to the business at hand, have intruded into the foreground *as* the business at hand, to be dealt with as an interactional topic before being again relegated back to the tacit background. The sound has been integrated into the situational setting as a mutually acknowledged un-remarkable aspect of that setting. But what has occurred here has been a little wobble in the mutual situational ontology, where all that is solid and unquestionably known about the situation, such as the chairs the situational members are sitting in, the walls of the room, and the understandings of the parts each other plays in the situation – the ground, in other words, that allows the situated business to proceed – recedes, and the unknown (unTypified) element emerges to be dealt with in foreground as something to be mutually agreed upon and Typified from their different perspectives as appearing sufficiently the same to both (as two people seated at opposite sides of a table will see two different aspects of a cup, but agree, for all practical purposes, that it is the same cup). This occurs, but we have suggested that there is an ‘oddness’ to the response at 72. Here, the interpretive eye of the analyst intrudes: what grounds do we have for claiming oddness?

In their only co-authored article, Garfinkel and Sacks make the case for sociological enquiry based on ‘members methods’. The notion of ‘member’, they claim, is ‘the heart of the matter’. They do not use the term to refer to a person, but to ‘mastery of natural language’, which itself means ‘to be engaged in the objective production and objective display of common-sense knowledge of everyday activities as observable and reportable phenomena’ (1970, 163).

In saying that something feels ‘odd’ about the response at line 70, and in the absence of the interviewer having oriented to the response as odd, we do so not as language analysts but as natural language members reflexively ‘noticing’ a glitch in expectation at the level of first-order meaning activity. In excavating that anomaly – by saying, for instance, that BF appears to exceed his warrants here by ‘granting pass’ to the noise – we move to practice second-order meaning-making. This inference is produced upon an imaginative projection of what would *not* have seemed odd here: BF simply ignoring the noise as part of a busy hospital setting, or else accepting at 72 the interviewer’s formulation (‘*oh, ok*’) would not have seemed odd, nor would the interviewer ‘granting pass’ to the noise as situationally appropriate.

In other words, the interviewer has situated membership roles that grant socially organized warrants to condone the noise as situation-appropriate and within the bounds of mundane occurrence, while BF’s warrants, organized relative to the interviewer’s, are correspondingly less.

By following chains of inference through and explicating expectations on the grounds of our own membership and our own natural language mastery, we use ethnomethodological self-reflection to demonstrate how the seeming institutional role reversal in the content of BF’s productions between lines 62 and 66 and taken up again between lines 73 and 76 is also apparent in the interactional detail of setting management.

### 5.2 What’s this thing you got goin’ ‘ere?

Now consider the following extract:**data extract 6, ‘what’s this thing?’**

This extract, with the same interlocutors as the last, occurs just before the sequence considered in data extract 1 within the same sequence of cognitive tests conducted by the second interviewer (I2). The interviewee (BF) had expressed reluctance from the start of this activity and appeared to actively subvert testing procedures. At one stage, when requested to answer ‘*in ordinary language… ordinary speech*’ after having used apparently non-sense words, he replied ‘*what you want me to answer it sensibly…*’, suggesting a strategic aspect to his engagement in the interview.

In lines 1332–1333, BF orients to a situational element in This-world mode of apparent negotiation of mutuality via explicit negotiation of *what-is-it-that-this-thing-is?* – offering, in turn, a candidate formulation (that the thing might be a recording device).

The first interviewer, who has had more time interacting with BF up to this point, responds to the problematization of the situational element and confirms the candidate formulation at 1334 with a follow-up elaboration—‘*yeah it’s just to show that it’s working’*. Mutual orientation is not immediately achieved, however, which leads to interactive trouble up to the point where BF rejects the interviewer’s situational interpretation at 1345 and then explicitly states at 1349, ‘*no I do not believe ya*’. As shown in extract 8, this is followed by a 0.5-s gap before I1 initiates what sounds like an abandoned response. Silence and *non-response* [strategies that Goffman (1969, p. 371) described as ‘damping’] then seem to be adopted by the interviewer, as the sequence between 1353 and 1361 shows, until the interactive trouble is resolved enough for the institutional business-at-hand to resume, and the second interviewer to return to the testing format:**data extract 7, ‘no I don’t believe ya’**

An entire situation is structured in myriad ways such that its members respond and orient to it meaningfully as a matter of course and as a matter of coordinated activity. Mostly, this occurs tacitly, as a mode of common-sense orientation to a shared life-world. As mentioned, this includes generic modes of orientation such as orienting to chairs in a room as meaningful organized elements by *acting* toward them as chairs (and sitting in them), to walls of a room *as* walls (demarcating social spaces, organizations, and activities), but this also funnels down to progressively more specialized modes of orientation – orienting to a hospital setting *as* a hospital setting, structured by certain mutual expectations of appropriacy, and not, for instance, orienting to it as a football stadium; and orienting to an interview situation *as* an interview situation, organized into elements, such as situational membership roles, expectation of outcomes (the gathering of records), and certain technical components, such as technologies of record-gathering.

All of these background organizations represent structural world knowledge held in common – 'That-world’ knowledge – that informs patterns of relevancy in the immediate interactional situation in the form of membership expectation. In That-world, dogs chase cats; in This-world, we see a cat chase a dog, and the discrepancy registers with a glitch of surprise. In being admitted to a hospital, a patient-member to its institutionally structured situations will have certain expectations in common with other patient-members as to how nurse-members, doctor-members, and administrator-members might act, as well as expectancy sets of appropriate response.

In talking of expectations, Tannen (1993) reminds us that we are talking of frames. We might say that this loose background of progressively nested frames and settings is organized in any given situation into an entire taken-for-granted gestalt even before the business-at-hand is entered into, and at the precise moment of the unfolding of the business-at-hand, it constitutes a perennially up-for-negotiation but nonetheless stable common-world for all parties to the situation.

It is *this* organization that we are talking about as ‘This-world’. This-world is an entire gestalt. It is structured, at any given moment, in terms of the organized absences of That-world, organizations of Type, and Typified relations. Disturb just one of these elements, and the edifice is discombobulated. We simply do not see this organization until it is transgressed. This background organization is the massive achievement that BF has already accrued before his foregrounding of the situational element of the recording device as the thread to pick apart, ambiguating the situation. A device for recording information in an interview setting that is itself situated within a hospital setting might flip, in an instantaneous aspect-switch, to seem a medical record-taking device. It is *this* aspect of This-world – the recording device – which has risen up, looming large, derailing the organized activity. In this sense, the recording device stands as a synecdoche to its specific embedding situatedness. If the ‘recording device’ should fail to hold firm to its function, then the entire situation – the ‘interview situation’ – is called into question in the same way. The doubt cast upon ‘what-it-is-that-this-thing-is’ casts into doubt ‘what-it-is-that-is-happening-here’ – the contract of shared action, grounded in the indubiety of the natural attitude, is disrupted.

The mode of BF’s problematizing now becomes of interest: ‘*what maths can you work out from it’*, ‘*is that mapped to a heart rate monitor*’, ‘*if you are graphing it’, ‘I wanna know what you can actually work out from it*’. His concern is about the translation from This-world contingency to more distal modes of account – to consolidation in That-world interpretations (over which he holds no authority), and even, bearing in mind Husserl’s critique of the post-Galilean ‘mathematization of nature’ (Moran, 2012), about the imposition of a The-world totalizing interpretation on his body (or self) as situated object. It is worth bearing in mind here Mishler’s (1984) distinction between the ‘the world of medicine’ and a patient’s lifeworld, frames which can come into conflict as a patient resists a reduction of the second to the first.

The irony here is that BF’s suspicions had some ground for justification. BF’s language *was* being sampled for scientific analysis and his meanings *were* being exported to another world in order to have second-order meanings constructed out of them; and at the time, there was a good chance that quantification of his languaging *would* in fact occur, that the embodied articulations of Me-world and This-world would be passed through the mathematical sieve to be mapped against the grid of The-world reckoning. His meanings, in other words, are taken out of his hands and subject to objectification.

In this context, it ought to be borne in mind that this excerpt occurred in the midst of a series of psychological tests. The critique that Husserl applied to ‘scientific psychology’ as the ground for the phenomenological epoché – that the objectifying, deanimating gaze of scientistic reckoning, when applied to the human subject, diminishes the valence of the experiential domain – may be relevant here. The totalizing nature of the scientistic world-frame (which permits of only one world) inherently affects the conception of the human dimension. BF is orienting to the ‘*recording (.) thing*’ as a translating device that does not only export his meanings to another (non This-) domain but mathematicises them, and he demands to be given access to these translations – ‘*I wanna know what you can actually work out from it*’. The interviewer, on the other hand, appeals to a common-sense mundanity of the device with a sequence of ‘*just*s (‘*just to show that it’s working’*, ‘*just the volume*’, and ‘*that’s just to m-...make*’) that function as markers of ‘non-remarkability’ working to de-thematise the recording device and relegate it to the background, taken-for-granted common world that does not warrant ‘tellability’ (Ochs and Capps, 2001).

## 6 Discussion

We began this article by asking how, as interactants, we distinguish between seemingly mad talk by people who are not diagnosed with mental illness and ‘hearably mad’ talk by people who are so diagnosed. This led to questions about how people come to inhabit a common world in the first place and an investigation into the constitution of the coordinated world of common-sense. It was observed that certain schools of psychiatry posit the root source of the wide range of *schizophrenia* symptoms to lie in a disturbance of common sense, which motivates the broadly ethnomethodological approach taken here. However, attempting to apply Conversation Analytic methods to recorded talk of diagnosees raised questions about the applicability of some of these methods to psychosis, and in addition, after Antaki and Wilkinson (2012), questions about their applicability to atypical populations in general.

An attempt to find common conceptual ground with psychiatric theory directed us to examine the phenomenological roots of ethnomethods in the works of Alfred Schutz and Edmund Husserl, which led to reconceptualizing *schizophrenia* in terms of a ‘world disturbance’. It was suggested, after Pollner (1974, 1987), that a study of world constitution in the natural attitude needed to occur from a stance that was itself distanced from the natural attitude, and this provided grounds for proposing the ‘manifold-world’ model that has been presented here in the manner of ‘breaching’, for analytic purposes, common-sense world assumptions.

Our conclusion is that consideration of atypical interactions compels us to take account of our own implicit normative world-frames, making clear the need for engaging more complex models of sense-making, such as the one that has been sketched here. We believe that finding ways to integrate such top-down modeling with the bottom-up rigor of traditional CA method might afford CA added explanatory leverage in cross-disciplinary applications.

In terms of implications for Conversation Analytic method, we suggest that the grounds of the second-order meaning-activities of interaction analysis should be recognized as lying in unavoidable biological activities of the analyst: the orienting of attention, the making of relevance judgments, and the interpretative activities of sense-making. Recognizing the biological ground of these second-order meaning-activities would help avoid the mistake of employing a sterilizing ‘scientism’ that would otherwise deanimate the objects under study by treating them in the manner of objects of natural science. To say this is to recognize that what is occurring in the study of talk-in-interaction is a form of life-world analysis. This point is acutely relevant when it comes to the study of interaction with *schizophrenia* diagnosees, as the dominant psychiatric framing of *schizophrenia* as ‘brain-disease’ risks just such a reduction of first-order meaning activity of diagnosees to (nothing more than) neurochemical imbalance. We would generalize the observation to include the study of all atypical populations.

To recognize the biological ground of meaning-activity is to recognize that any interaction analyst is irreducibly relevance-orienting and account-generating and reads observational data in light of these relevancy fields and reflexively generated organizing accounts. In other words, any attempts at bottom-up processes of observation in interaction analysis will unavoidably meet top-down processes of plausible model generation. We make these claims because the processes under description are inherent to our activities as biological organisms. These observations are consistent with general approaches of enactivism (Varela et al., 1991; Gallagher and Allen, 2018) as well as with recent collaborations between neurophysiology and philosophy, which fall under the broad rubric of ‘predictive processing’ or ‘predictive coding’ (Hohwy, 2013; Clark, 2015; Friston and Frith, 2015; Seth, 2021), where top-down modeling is seen to be ineluctably involved with attentional and relevance processing across all levels of meaning organization.

This brings the analyst once again within the analytic frame. We utilized normative membership reflexes as a potential source of information via ethnomethodological self-reflection and by paying heed to ‘hearable madness’ as well as ‘glitches’ in analyst-as-member expectation. Psychiatric readers might recognize such self-reflection as a cousin to counter-transference, and the interested reader is directed to Rumke’s (1990) writings on ‘praecoxfeeling’, where it is argued that *schizophrenia* diagnosis occurs via intersubjective processes, recommending clinician attention be paid to their own internal responses when interacting with a diagnosee in addition to searching for explicit external signs.

In line with Garfinkel’s unique adequacy requirement, it was seen necessary to explore the theoretical background of *schizophrenia* research, which included a critical examination of psychiatric constructs, including, most importantly, the *schizophrenia* construct itself. The resultant model we have proposed has implications in psychiatry for theoretical considerations on topics such as *schizophrenic* autism, double bookkeeping, disturbance of abstraction/concretism (Shimkunas et al., 1967), theories of disturbed indexicality (Crow, 2010), and insight attribution.

Exploring psychiatric theory led to an examination of phenomenological approaches to *schizophrenia*. In light of this, Garfinkel’s rejection of phenomenological method (Garfinkel et al., 1977) should be qualified as a rejection of Husserlian transcendence, and we suggest the ethnomethodological project be recognized as a methodological unfolding of Schutz’s proposal of a complementary (social) form of phenomenological enquiry. Considering this genealogy highlights the theoretically dense lineage that led up to CA’s ultimate abjuration of theory to focus on method. We found it both necessary and fruitful to exhume the historical connection and conceptual links between CA and the Husserlian project and suggest that if CA should cut itself off from its theoretical sources, it risks separating itself from a font of renewal, in danger of becoming a technical exercise in cataloging that fails to establish footholds of relevance in other domains.

On this note, we believe that possibilities for dialectic engagement between Conversation Analysis and psychiatry are untapped. In psychiatry, for example, Conversation Analytic methods might prove useful in the training of clinicians to identify structures of intersubjectivity, and in language-in-interaction research, the general topic of interaction with atypical populations remains under-serviced. Institutional psychiatry, which has been dealing with atypical interlocutors since its inception, will likely have developed idiosyncratic norms of interaction which – bearing in mind Jakobson’s injunction to study breakdown in order to gain a better understanding of function – might prove of inherent interest to language researchers.

In summary, we present the model of a five-world manifold as a motivated choice that has its ground in various forms of phenomenological and sociological theory. What is being suggested here is that the achievement of the one-world-in-common among and between members of language communities represents an achievement of common sense coordination. It is the *supreme* achievement of common sense and represents a lived commitment to the social world. It is a deeply habituated background assumption of the natural attitude that the language analyst themself is committed to in the mundane common-sense mode of acting and interacting in the world. It represents a *model of reality* that is already in operation as a background assumption behind day-to-day affairs. Introducing a five-world model, or meaning manifold, as has been done here, does not so much represent a theoretical imposition upon a tabula rasa but rather displaces the unconscious model of one-world bearing the load of all concrete linguistic reference that is already in operation within the natural attitude.

## Data availability statement

The original contributions presented in the study are included in the article, further inquiries can be directed to the corresponding author.

## Ethics statement

The studies involving humans were approved by the University of Melbourne Humanities and Applied Sciences Human Ethics Sub-Committee. The studies were conducted in accordance with the local legislation and institutional requirements. Written informed consent for participation was not required from the participants or the participants’ legal guardians/next of kin because data used in this study was collected in a prior, independent study where informed consent was negotiated.

## Author contributions

All authors listed have made a substantial, direct, and intellectual contribution to the work and approved it for publication.

## Footnotes

^1^A word on nomenclature: the term ‘schizophrenia’ has been problematized here and its italicization is intended to foreground social processes of diagnosis and construct-formation. To pursue this focus, we additionally refrain from talking of interview subjects as ‘patients’ or ‘people with schizophrenia’, but instead refer to them as ‘diagnosees’. In line with social movements to destigmatize mental health (e.g., “mad pride,” see Rashed, 2023), the use of ‘mad’ is not similarly italicized, with the word intended to describe pre-diagnostic organizations of non-normativity. A more detailed consideration of this usage will be presented in section 4.1.1.

^2^The phenomenological psychiatrist Jaspers (1963) positioned psychiatry at the confluence of these two approaches, although famously insisted that core aspects of *schizophrenia* were ‘un-understandable’, and thus closed to being approached via *verstehen* methods of empathic understanding.

^3^Any non-standard transcription markings can be found in appendix.

^4^For a consideration of such a general distinction as frame-conflict in medicine, see Mishler (1984).

^5^Note that this is not simply a reinstatement of the object-world, but involves social co-ordination as necessarily constitutive of the world. Schutz describes this by a series of postulates that guarantee a ‘reciprocity of perspectives’ (1962a, 315–316), whereby actors “maintain the belief that others perceive reality as we do...(such that)... if we were to change places with others our perception of reality would remain the same...” (Stubblefield and Murray, 2002, p. 151).

^6^We use ‘Type’ — and capitalize it – after Schutz (1962c), rather than the more cognitively loaded ‘category’. Nonetheless, they perform more or less the same function here.

^7^‘Autism’, as used here, is different to ‘autism’ as commonly applied to Autism Spectrum Disorders. The use of the term to describe key features of *schizophrenia* originated with Bleuler (Fusar-Poli and Politi, 2008), predating application of the term to developmental anomalies.

^8^A similar ‘enigmatic pointing’ can be seen with the indexicals bracketing the ‘mad talk’ in data extract 1 in lines 58 and 72.
