# Supplementary material for: A conversation analytic approach to schizophrenic interaction: methodological reflections on disruptions of the common-sense world
Source: Front Sociol. 2023 Dec 19;8:1223186. doi: 10.3389/fsoc.2023.1223186 (PMC10805113; doi:10.3389/fsoc.2023.1223186)
Supplement: Supplementary file 1 [file Data_Sheet_1.docx]

# Transcription Conventions

Adapted from DuBois, revised transcription conventions—DT2 (Du Bois, 2006)

| Primary accent | yourself |
| --- | --- |
| Secondary accent | yourself |
| Forte | WORD |
| Piano (softer) | ^o^word^o^ |
| Glottalised word | w%ord |
| micropause/missed beat | (.) |
| pause, short | .. |
| pause, long | ... |
| pause, timed | (1.0) |
| lag, prosodic lengthening | : |
| disfluent/nonstandard tie | _x_beat_x_ |
| truncated intonation unit | - |
| uncertain hearing | (word) |
| unintelligible | (###) |
| sub/unformed vocalisation | {_##_/##} |
| sub/unformed vocalisation | {_tht_/tht} |
| indeterminate phoneme | (b), (m/b) etc. |
| latching | = |
| rising intonation (pitch jump) | ￪ |
| falling intonation | ￬ |
| falling terminal (final) | . |
| falling terminal (continuing) | ; |
| appeal final/continuing | ?/?, |
| line of interest | ⟶ |
| faster | > word < |
| slower | < word > |
| fluent run | *words in italics* |
| inbreath | (H) |
| outbreath | (Hx) |
| laugh pulse (word ex/internal) | @ / wo@rd |
| transcriber’s description/comments | ((coughing?)) |
| Intertextual footnote (ignore) | Syndrome^2^ |
